# Relationship of microvascular density on histological and immunohistochemical features in endometrioid adenocarcinomas of the uterus: experimental study

**DOI:** 10.1097/MS9.0000000000000939

**Published:** 2023-06-13

**Authors:** Mykola Lуndіn, Olga Kravtsova, Kateryna Sikora, Yulia Lуndіna, Yana Sikora, Wireko Andrew Awuah, Mohammad M. Hasan, Toufik Abdul-Rahman, Vladyslav Sikora, Nataliia Hyriavenko, Anatolii Romaniuk

**Affiliations:** a Sumy State University, 2, Rymskogo-Korsakova street, Sumy, Ukraine; b Department of Biochemistry and Molecular Biology, Faculty of Life Science, Mawlana Bhashani Science and Technology University, Tangail, Bangladesh; c Department of Clinical and Experimental Medicine, University of Foggia, Via Napoli, 20, Foggia, Italy

**Keywords:** endometrioid adenocarcinoma, microvascular density, uterus cancer

## Abstract

The most common malignant tumors of the uterus are endometrioid adenocarcinomas (EA). Their prognosis depends on the qualitative characteristics of the neoplastic cells and their stroma. The neovascularization of EA tissues and level of microvascular density (MVD) influence tumor progression. Our study aims to establish the relationship between MVD in EA tissue and the histological and immunohistochemical features of tumors.

**Materials and methods:**

The authors studied 30 cases of endometrial ЕА and compared their histological and immunohistochemical characteristics with the MVD of tumor tissues.

**Results:**

Our study indicated that MVD in EA tissue depends on the grade of the tumors and their FIGO stage. Increased MVD was correlated with a depression of E-cadherin and PR expression and enhanced expression of VEGF and Ki-67. MVD enhancement during VEGF overexpression is a manifestation of the functional activity of these proteins. The increase in MVD was accompanied by more frequent metastasis of the EA to the lymph nodes.

**Conclusion:**

EA progression is accompanied by qualitative and quantitative variations of parenchymal and stromal patterns of tumors. Dedifferentiation of EA leads to overexpression of VEGF, which becomes diffuse in tumors cells, resulting in an increase of adenocarcinomas’ MVD and their metastatic potential. Correlations between histological and immunohistochemical features of EAs indicate the synchronicity of the occurrence and progression of morphological and immunological anaplasia, which can be used in predicting the course of the disease.

## Introduction

Highlights

- The most common malignant tumors of the uterus are endometrioid adenocarcinomas (EA). Its progression is accompanied by qualitative and quantitative variations of parenchymal and stromal patterns of tumors.
- The neovascularization of endometrioid adenocarcinoma tissues and the level of microvascular density influence tumor progression.
- Dedifferentiation of EA leads to overexpression of VEGF, which is diffuse in cells.
- Correlations between histological and immunohistochemical features of EA indicate the synchronicity of the occurrence and progression of morphological and immunological anaplasia, which can be used in predicting the course of the disease.

The most common malignant tumors of the uterus are endometrioid adenocarcinomas (EA), which account for about 70–80% of all endometrial cancers^1^. Classified as type I endometrial carcinomas, they are predominantly sensitive to hormone therapy^2^. Depending on the area occupied by solid growth, EAs are divided into the categories of well-differentiated (grade 1), moderately differentiated (grade 2), and poorly differentiated (grade 3). The presence of grade 3 nuclear pleomorphism can increase the tumor grade by one position^3^. Among the proven prognostic markers, the most important indicators are the stage of the disease, the patient’s age, the tumor’s histological grade, as well as expression of estrogen receptors (ER), progesterone receptors (PR), the percentage of Ki-67-positive tumor cells, and the expression of mutant p53 oncoprotein^2^.

The course of malignant tumors can be influenced not only by the qualitative characteristics of neoplastic cells but also by their stroma. In recent decades, attention has increasingly turned to the neovascularization of EA tissues and the resulting effect on tumor progression^4–12^. An objective indicator of this process is the level of microvascular density (MVD), which in EAs significantly exceeds that of nontumour tissue^7,9,10^. The effect of MVD on the overall survival rate^5,7,8^, as well as the presence of lymphovascular invasion^4,7,8^, myometrial invasion^8,^ and tumor metastasis^7,8^ has been described. Higher MVD levels were observed in less differentiated EAs (grades 2 and 3)^4,6,9^. Relationships also exist between the expression of receptors by tumor cells and the neovascularization of EA tissue^4–6^: MVD growth was observed alongside overexpression of vascular endothelial growth factor (VEGF)^5,6,12^, hypoxia-inducible factor 1α, and glucose transporter-1^12,^ as well as in tumors negative for ER, PR, or both^4^. In uterine carcinosarcomas, the epithelial component had been shown to have a higher MVD than the stromal^11^.

Our study aims to establish the relationship between MVD in EA tissue and histological features of tumor tissue; the expression of ER, PR, Ki-67, mutant p53, E-cadherin, and VEGF by tumor cells; and its effect on the diagnostic accuracy and metastatic potential of the carcinoma. Carcinoma progression of the reproductive system organs (differentiation and metastatic spread) was accompanied by the suppression of ER, PR, and E-cadherin expressions^13,14^. An increase in tumor grade was related to increased Ki-67, p53, and VEGF expression^13–15^. Data on their combined effect on the MVD of EA was limited.

## Materials and methods

### Tissue collection

All patients were treated at the regional oncology center. We studied 30 cases of endometrial cancer. In order to exclude the influence of the histological type of endometrial carcinomas on the morphological and immunohistochemical parameters of tumors, we studied only endometrioid endometrial adenocarcinomas. Other histological types of endometrial adenocarcinomas (clear cell, serous, squamous, and others) and other malignant tumors (sarcomas) were excluded from the study. The selection of tissue samples was performed randomly, regardless of the patient’s age and FIGO stages. One of the study’s limitations was the inability to include cases of uterine adenocarcinomas with FIGO stages IV in the study group, as they were not found in the archive (they were not found during sample selection). All tumors were classified according to WHO recommendations. Patients provided their written informed consent for tissue investigation. The presented study is reported in accordance with the STARD criteria (Standards for Reporting of Diagnostic Accuracy Studies). The Bioethics Commission of the University approved the experimental protocol (no. 14 of 11.03.2020).

### Histology and Immunohistochemistry

For histological examination, we created 5 µm thick sections from the paraffin-embedded blocks of tissues and applied them to SuperFrost (Thermo Fisher Scientific; Waltham) microscope slides, which were subjected to standard deparaffinization with xylene and rehydration in decreasing ethanol concentrations. Then, slides were stained with hematoxylin and eosin by standard methods.

IHC and protein visualization were performed according to the manufacturer’s recommendations. We created 4 µm thick sections from the paraffin-embedded block of tissues, applied them to SuperFrost (‘Thermo Scientific’) microscope slides, and subjected the slides to standard deparaffinization in two portions of xylene and rehydration in decreasing concentrations of ethanol. Antigen unmasking was performed by heat-induced epitope retrieval with citrate (pH 6.0, 97^0^С, 30 min) in a water bath ‘WB-4’ (Ukraine). Receptor imaging was conducted using the UltraVision Quanto Detection System HRP Diaminobenzidine Chromogen (Thermo Scientific) at room temperature, which involves blocking endogenous peroxidase activity with hydrogen peroxide (10 min), conjugation with primary antibodies (30 min), blocking nonspecific background signals using ‘Ultra V block’ (5 min), and amplifying the reaction with ‘Primary Antibody Amplifier Quanto’ (10 min). Tris buffer (pH 7.3) was used to wash the samples between all steps of immunohistochemical staining (3 times for 10 min). The final visualization was done with diaminobenzidine under a microscope (the expression patterns were brown). The reaction was terminated by immersing the slides in distilled water. Cell visualization was performed using Mayer’s hematoxylin. In the immunohistochemical study, we used rabbit monoclonal antibodies to ER (clone SP1), PR (clone YR85), Ki-67 (clone SP6), and E-cadherin (clone 67A4) (‘Thermo Scientific’); mouse monoclonal antibodies to p53 (clone SP5) (‘Thermo Scientific’); rabbit polyclonal antibodies to VEGF (‘Thermo Scientific’); and mouse monoclonal antibodies to CD31 (clone 1A10) (‘Bio-Rad’).

### Morphometry

Three pathologists independently analyzed histological and immunohistochemical staining evaluations. If they did not reach a consensus regarding the results, we sought help from other specialists in this field or excluded samples from the study group. To determine the pattern of E-cadherin and VEGF expressions, we used a simplified three-tiered scoring system: ‘-’ for negative expression, ‘+’ for low expression, ‘++’ for moderate expression, and ‘+++’ for strong expression (0, 5–5, 26–50, and >50% positive cells, respectively). Only the aberrant p53 nuclear expression pattern was recorded as positive or negative staining. For expression of ER, PR, and Ki-67, we recorded the percentage of receptor-positive cells among the total number of tumor cells. According to generally accepted recommendations^7,^ MVD was determined in three fields of view under a magnification of ×200. The average value of the three most vascularized fields of view (‘hot spots’) was considered to be the MVD. Positive patterns in the slides were considered structures with CD31 expression and lumens (full-fledged vessels) and groups of cells with CD31 expression that did not form lumens. All histopathological examinations were done using the Carl Zeiss Primo Star microscope with a Zeiss AxioCam ERс 5s digital camera and the ZEN 2 (blue edition) software package.

### Statistical analysis

Data processing was carried out using the GraphPad Prism® statistics software package, version 6.0. We calculated the arithmetic mean (M) and SD. The estimation of the differences between the comparable indicators was carried out using Mann–Whitney (u). The significance of differences between the three groups was determined by one-way analysis of variance (ANOVA) with the Bonferroni a posteriori test. The detection and evaluation of the correlations between the indicators was carried out by the nonparametric Spearman correlation coefficient (r). A *P*-value of 0.05 (95% CI) was considered statistically significant.

## Results

### Characteristics of the patient groups

**Figure 1:**
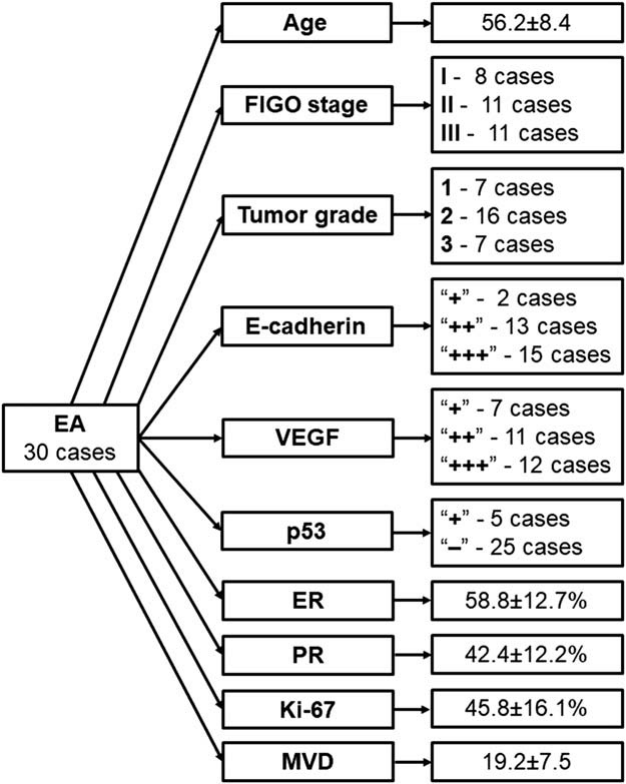
STARD diagram to report the flow of participants through the study.

The average age of EA patients was 56.2±8.4 years. According to the FIGO classification, among the 30 studied cases, eight corresponded to stage I and 11 to stage II and III (Fig. 1 and Table 1). We found significant differences between the groups only in terms of PR expression by tumor cells (a lower percentage of receptor-positive cells in stage II–III tumors (F=4.2, *P*=0.026)) and MVD carcinoma (higher MVD in stage II–III tumors (F=10.33, *P*=0.0005)).

**Table 1:** Characteristics of patient groups by FIGO stage.

G	n	FIGO	Age (average)	^***^E-cadherin	^*^VEGF	^**^ER (average)	^***^PR (average)	^***^Ki-67 (average)	^**^p53	^**^MVD	Mts
1	7	1–4/7	57.3	1–0/7	1–4/7	65.6	51.0	25.9	0/7	14.6	1/7
		2–2/7		2–0/7	2–2/7						
		3–1/7		3–7/7	3–1/7						
2	16	1–3/16	56.2	1–0/16	1–3/16	53.3	44.6	45.7	1/16	18.1	6/16
		2–7/16		2–8/16	2–7/16						
		3–6/16		3–8/16	3–6/16						
3	7	1–1/7	55.0	1–2/7	1–0/7	43.4	29.0	66.1	4/7	26.3	4/7
		2–2/7		2–5/7	2–2/7						
		3–4/7		3–0/7	3–5/7						

There is a significant difference between groups of patients according to ANOVA.

* *P*<0.05.

** *P*<0.01.

*** *P*<0.001.

**Figure 2:**
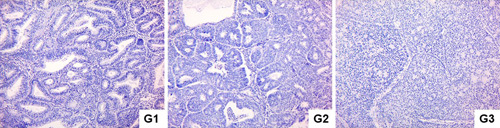
Different histological grades of EA. Hematoxylin and eosin staining. Magnification ×100.

During the study of histological samples, we found 7 of them to be grade I, 16 grade II, and 7 grade III. The level of EA differentiation depended on the area of the tumor with solid patterns and nuclear atypia, with grade 1 tumors being composed of less than 5% of solid growth and having mild nuclear atypia, grade 2 tumors having an area composed of 6–50% solid growth and showing moderate nuclear atypia, and grade 3 tumors being composed of more than 50% solid growth by area and showing severe nuclear atypia (Fig. 2).

Additionally, we found differences between EAs of different histological grades (Table 2). High-grade tumors (grades II and III) demonstrated depressed expression of E-cadherin (F=14.8, *P*<0.0001), ER (F=7.96, *P*=0.0019), and PR (F=10.25, *P*=0.0005). Carcinoma dedifferentiation was accompanied by increased expression of VEGF (F=4.56, *P*=0.02), percentage of Ki-67-positive tumor cells (F=41.9, *P* < 0.0001), and MVD (F=6.34, *P*=0.0055). High-grade tumors also more frequently had aberrant expression of p53 (F=7.71, *P*=0.0022).

**Table 2:** Characteristics of patient groups by tumor grade

FIGO	n	G	Age (average)	E-cadherin	VEGF	ER,% (average)	^*^PR, % (average)	Ki-67, % (average)	p53	^***^MVD	Mts
I	8	1–4/8	54.1	1–1/8	1–3/8	57.6	49.1	41.9	1/8	14.9	0/8
		2–3/8		2–2/8	2–3/8						
		3–1/8		3–5/8	3–2/8						
II	11	1–2/11	59.1	1–0/11	1–3/11	53.4	44.9	42.6	3/11	16.0	0/11
		2–7/11		2–7/11	2–2/11						
		3–2/11		3–4/11	3–6/11						
III	11	1–1/11	54.8	1–1/11	1–1/11	50.5	36.0	51.9	1/11	25.5	11/11
		2–6/11		2–4/11	2–6/11						
		3–4/11		3–6/11	3–4/11						

There is a significant difference between groups of patients according to ANOVA.

* *P*<0.05.

** *P*<0.01.

*** *P*<0.001.

### Results of the immunohistochemical assay

**Figure 3:**
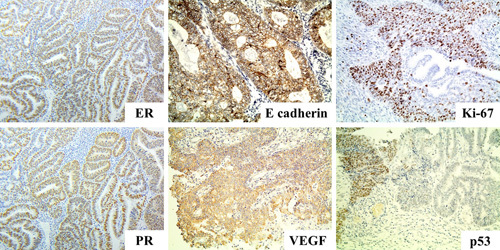
EA tissues with expressions of ER, PR, E-cadherin, VEGF, Ki-67, and mutant p53, detected by immunohistochemistry. Magnification ×200.

An immunohistochemical study found that E-cadherin expression was weak in 6.7% of EA tissue cases, moderate in 43.3%, and strong in 50.0%. VEGF expression was observed to be weak in 23.3% of samples, moderate in 36.7%, and strong in 40.0%. EA tissue revealed diffuse granular localization of VEGF in the cytoplasm of tumor cells with a tendency to perinuclear apical localization in well-differentiated tumors. Stromal leukocytes and vascular endothelium served as active internal quality control in the immunohistochemical study. Expressions of ER, PR, and Ki-67 were detected in all tumor samples, averaging 58.8±12.7, 42.4±12.2, and 45.8±16.1%, respectively. The presence of aberrant expression of p53 was detected in only 16.7% of cases. Examples of positive expressions of the above proteins are provided in Figure 3.

### MVD study in EA

**Figure 4:**
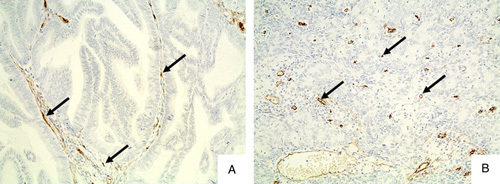
EA tissue with CD31 expression. EA samples with low and high vascularization (vessels display a positive DAB signal marked by an arrow). Magnification ×200.

An immunohistochemical study of CD31 expression revealed varying degrees of neovascularization in EA tissue (Fig. 4). In one field of view of the microscope at a magnification of ×200, we found from 5 to 37 vessels. The average MVD was 19.2±7.5. As noted earlier, more aggressive (grade 2 and 3; F=6.34, *P*=0.0055) and larger (stage 2 and 3; F=10.33, *P*=0.0005) tumors had higher MVD. The MVD was not related to the age of the patients (r=–0.002, *P*=0.99).

### Correlation analysis

Our study demonstrates a positive correlation between the expression of ER and PR (r=0.37, *P*=0.046). Negative correlations existed between the expression of ER and Ki-67 (r=–0.5, *P*=0.0045), PR and Ki-67 (r=–0.62, *P*=0.0003). EA tissue with expression of mutant p53 had lower E-cadherin expression (u=22.5, *P*=0.014) and higher VEGF (u=9.0, *P*=0.049) and Ki-67 (u=14.0, *P*=0.0074) expressions.

**Figure 5:**
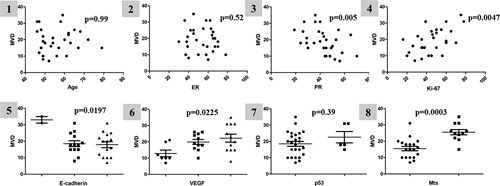
Study of the relationship between MVD; patient age; the expression of ER, PR, Ki-67, E-cadherin, VEGF, p53; and EA metastasis. The presence of statistical reliability indicates *P* < 0.05. 1–4 Spearman correlation coefficient analysis, 5–6 analysis of variance (ANOVA), 7–8 Mann–Whitney analysis.

In addition, we found that MVD depended not only on the morphological characteristics of carcinomas (their grade) but also on the receptor profile of neoplastic cells (Fig. 5). Higher MVD was observed alongside depression of E-cadherin expression (F=4.56, *P*=0.0197) and overexpression of VEGF (F=4.38, *P*=0.0225). At the same time, we observed an increase in MVD alongside suppression of PR expression (r=–0.5, *P*=0.005) and increased cell proliferative activity (r=0.5, *P*=0.0047). A noteworthy finding is that carcinomas that metastasized to the lymph nodes had significantly higher MVD (u=20.5, *P*=0.0003) than nonmetastatic carcinomas.

Among the histological and immunohistochemical characteristics of EAs, only PR expression demonstrated a relationship to their metastatic potential: EAs with lymph node metastases had lower levels of PR expression (u=43.0, *P*=0.0086).

## Discussions

Although malignant tumors of the uterine body rank 15th in the structure of cancer in the world (2.2% of all cases), they are a common cause of death among women^16^. The most common uterine cancer variant is EA^1^. The prognosis of EA depends on many factors; the most important of which are the stage of the disease and the histological tumor grade^2^. In addition, a proven relationship exists between the receptor profile as of intact cells as tumor cells, tumor microenvironment, and cancer progression^2,13–15,17–19^. The features of the stromal component also affect the course and metastasis of malignant tumors^4,5,7,8,20^. MVD, one of the critical indicators of the metastatic potential of carcinoma, depends on several histological and immunohistochemical features of tumors^5,6,12^.

Our study indicated that the angiogenesis intensity in EA tissue depends on the grade of the tumors and their FIGO stage. We observed an increase in MVD during tumors’ dedifferentiation and spread. MVD was unrelated to the age of the patients. The receptor profile of tumor cells had a pronounced effect on the vascularization of EA tissues. Increased MVD was correlated with a depression of E-cadherin and PR expression and enhanced expression of VEGF and Ki-67. The relationships between the level of tissue vascularization and the expression of PR, E-cadherin, and cell proliferative activity was observed in the synchronous changes in these characteristics during the progression and dedifferentiation of EAs. This is due to the lack of functional bridges between these proteins and tissue angiogenesis. At the same time, we also do not rule out the presence of a common influence on their variability, dependent, for example, on other proteins.

**Figure 6:**
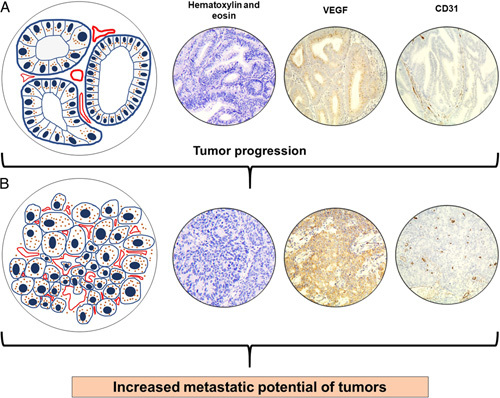
Scheme of MVD growth in EA tissue during cancer progression (grade change) and variability of receptor profile of tumor cells. (A) Low MVD in low-grade EA. Tumor cells with predominantly perinuclear (on the apical side) location of VEGF (marked in pink in the diagram). Low probability of VEGF entering extracellular tissues. (B) High MVD in high-grade EA. Tumor cells with diffuse VEGF localization. Higher possibility of VEGF getting into extracellular tissues with stimulation of angiogenesis.

MVD enhancement during VEGF overexpression is a manifestation of the functional activity of these proteins, which, after release from neoplastic cells, stimulate the formation of new vessels in and around the tumor, causing endothelial cell proliferation and migration as well as degradation of the extracellular matrix^21^. The result is an increase in the supply of oxygen and essential substances to the carcinoma, stimulating tumor growth. In addition, the newly formed vessels possess increased wall permeability, which becomes the gateway for the tumor’s lymphogenic and hematogenous metastasis. We demonstrated in our study that the increase in MVD was accompanied by more frequent metastasis of the EA to the lymph nodes. The relationships between tumor progression, MVD enhancement, and carcinoma metastasis are provided in Figure 6.

Our results also confirmed previously published data on the effect of the grade of the EA on the expression of prognostically essential proteins^22,23^. Carcinoma dedifferentiation was accompanied by E-cadherin, ER, and PR expression inhibition and increased expression of VEGF, mutant p53, and Ki-67. In addition, we found a positive correlation between the expression of ER and PR; negative correlations between the expression of ER and Ki-67, PR and Ki-67. This indicates the synchrony of morphological and immunological anaplasia with the progression of EA and the ability to predict the course of the disease using histological and immunohistochemical diagnostic accuracy studies (STARD statement) of biopsy material.

We found a difference in the expression of receptors in different FIGO stages only for PR. EAs with lymph node metastases also had lower levels of PR expression. In our opinion, the presence and level of PR expression is a more prognostically important indicator. This has been reported in previous studies; however, those have focused on breast cancer rather than endometrial adenocarcinoma^24–26^.

## Conclusions

Dedifferentiation of EA leads to overexpression of VEGF, which becomes diffuse in tumors cells, resulting in an increase of adenocarcinomas’ MVD and their metastatic potential. Higher MVD was observed alongside depression of E-cadherin and PR expression, overexpression of VEGF and increased tumor cells proliferative activity. Correlations between histological and immunohistochemical features of EAs indicate the synchronicity of the occurrence and progression of morphological and immunological anaplasia. Therefore, the features of proteins expression can be used in predicting the course of the disease and its spread.

## Ethics statement

Ethical approval based on the Declaration of Helsinki with the registration of this study was provided by the Bioethics Commission of the Academic and Research Medical Institute of Sumy State University, Sumy, Ukraine (Certificate number of 10/03, from 11 March 2020).

## Patient consent

Written informed consent was obtained from the patient for the publication of this case report and accompanying images. A copy of the written consent is available for review by the Editor-in-Chief of this journal on request.

## Sources of funding

This study was supported by the Ministry of Education and Science of Ukraine under Grant № 0121U100472 and Grant № 0123U100111.

## Author contribution

M.L., O.K., K.S., Y.L., N.H., M.M.H., V.S., and A.R. were involved in the conceptualisation and design of the manuscript; M.L., O.K., K.S., Y.L., Y.S., A.W.A., T.A.R., M.M.H., N.H., and V.S. participated in data curation and investigation; M.L., O.K., K.S., Y.L., Y.S., A.W.A., T.A.R., M.M.H., N.H., and V.S. performed data analysis, formal analysis, and statistical analysis; M.L., O.K., K.S., Y.L., A.W.A., T.A.R., M.M.H., N.H., V.S., and A.R. drafted the manuscript; M.L., V.S., and A.R. reviewed and edited the manuscript. All authors critically revised the final version of the manuscript and had responsibility for the final content.

## Conflicts of interest disclosure

The authors declare that they have no financial conflict of interest with regard to the content of this report.

## Research registration unique identifying number (UIN)

Research registration unique identifying number (UIN) Name of the registry: NA. Unique identifying number or registration ID: NA. Hyperlink to your specific registration (must be publicly accessible and will be checked): NA. I would like to bring to your attention that our manuscript was submitted to a journal before registration of the study became necessary. The review process in your journal has already taken approximately 8 months. Our research on archival blocks of tumors was conducted between 2020 and 2021. That is why registering the study, which was already completed two years ago, is a relatively tricky procedure. Moreover, we have encountered difficulties with registering the research on the paid database, as the associated paid services are currently unaffordable for Ukraine due to the blockade of our international financial operations resulting from the war. At the same time, this study successfully passed all stages of ethical checks at the regional and national levels, as indicated above. Ethical approval based on the Declaration of Helsinki with the registration of this study was provided by the Bioethics Commission of the Academic and Research Medical Institute of Sumy State University, Sumy, Ukraine (Certificate number of 10/03, from 11 March 2020). I respectfully request that you consider our situation and assist us in finding a solution.

## Guarantor

Prof. Anatolii Romaniuk, MD, DMSc, Head of the Department of Pathology, Sumy State University, Sumy, Ukraine. E-mail: pathomorph@gmail.com ORCID: 0000-0003-2560-1382

## Data availability statement

The data of current study are publicly available.

## Provenance and peer review

Not commissioned, externally peer reviewed.
